# “OCTOPUS”: An Intelligent Tool for Assisted Multidisciplinary ORL Oncology Meetings—Preliminary Study

**DOI:** 10.1002/oto2.64

**Published:** 2023-07-12

**Authors:** Naouar Ouattassi, Anas Azdad, Hamza Debaghi, Arsalane Zarghili, Mohamed Nouredine El Amine El Alami

**Affiliations:** ^1^ ENT, Head and Neck Surgery Department, Anatomy Surgery and Anesthesiology Laboratory Hassan II University Hospital Fez Morocco; ^2^ Computer Science Department, Intelligent Systems and Applications Laboratory, Science and Technic Faculty University Sidi Mohammed Ben Abdellah of Fez Fez Morocco

**Keywords:** cancer, MDMs, multidisciplinary oncology meetings, tumor boards

## Abstract

Current sessions of multidisciplinary team meetings (MDMs) have several limitations, including increased costs and duration, as well as limited discussion time for each case. To address these issues, we have developed a computer application called OCTOPUS, which helps manage the patients' list for MDMs. The application allows for the generation of automatic MDM decisions based on predetermined criteria filled in by the patient's doctor and algorithms that comply with the latest oncology guidelines from the National Comprehensive Cancer Network. These decisions must be validated by the MDM faculty members. In cases of nonagreement or complex cases, the application proposes a “face‐to‐face MDM.” The internal mailing system connects all members and allows for the request of a second opinion regarding pathology or imaging results. Initial results suggest that this ergonomic tool provides more flexibility in time management and improved uniformity in selecting cases that would most benefit from face‐to‐face MDMs.

In cancer management, universal guidelines are followed within multidisciplinary team meetings (MDMs), also known as tumor boards or multidisciplinary oncology meetings. These meetings aim to provide patients with the optimal health care plan and access to diverse clinical expertise to reduce reported inequalities in care.^1^, ^2^ However, meeting duration has been known to increase by up to 5 hours in some cases due to workload and staff shortages.^2^ Additionally, the time allowed to discuss each case is shortened, with a median duration of case review being reported at 2 minutes in some MDM meetings.^2^ This raises questions about whether these decisions align with the optimal decision‐making process intended for MDMs. Furthermore, studies have shown that current MDMs can be both time‐consuming and expensive.^3^

Our objective is to develop a reliable tool for therapeutic decision‐making in cancer cases and a more standardized method of selecting patients for in‐person MDMs, in order to improve the cost‐effectiveness and time management of these meetings.

## Methods

Approval from the Fez University Ethics Committee was not required for this study as it does not involve human data. We developed a web application named OCTOPUS that performs automatic processing of oncology cases and provides therapeutic proposals, which must be validated by MDM members.

### Study Design and Settings

OCTOPUS generates automatic therapeutic decisions based on predetermined criteria filled in by the patient's doctor and algorithms based on National Comprehensive Cancer Network's (NCCN) guidelines. A preliminary study was conducted on upper aerodigestive tract cancers, using available online data on cancer management according to NCCN's guidelines. The backend of the application is built on Laravel, a PHP Language Framework, and SQL Language. The front end relies on technologies such as jQuery, CSS, HTML, and Bootstrap, among others. The elaboration process went through 3 phases

#### Conceptualization of a Standard Cancer Patients' File

We noticed that therapeutic decisions in otolaryngology MDM files are based on a few factors such as tumor location, tumor, node, metastasis staging, tumor pathology, poor prognosis factors, whether surgery, chemotherapy, or radiation is feasible, and if there is a tumor relapse.

#### Implementation of Decisions' Diagrams

We established diagrams that summarize therapeutic plans for each sublocation of upper aerodigestive tract tumors according to the latest guidelines of the NCCN.

#### Ergonomic Sittings

**Figure 1:**
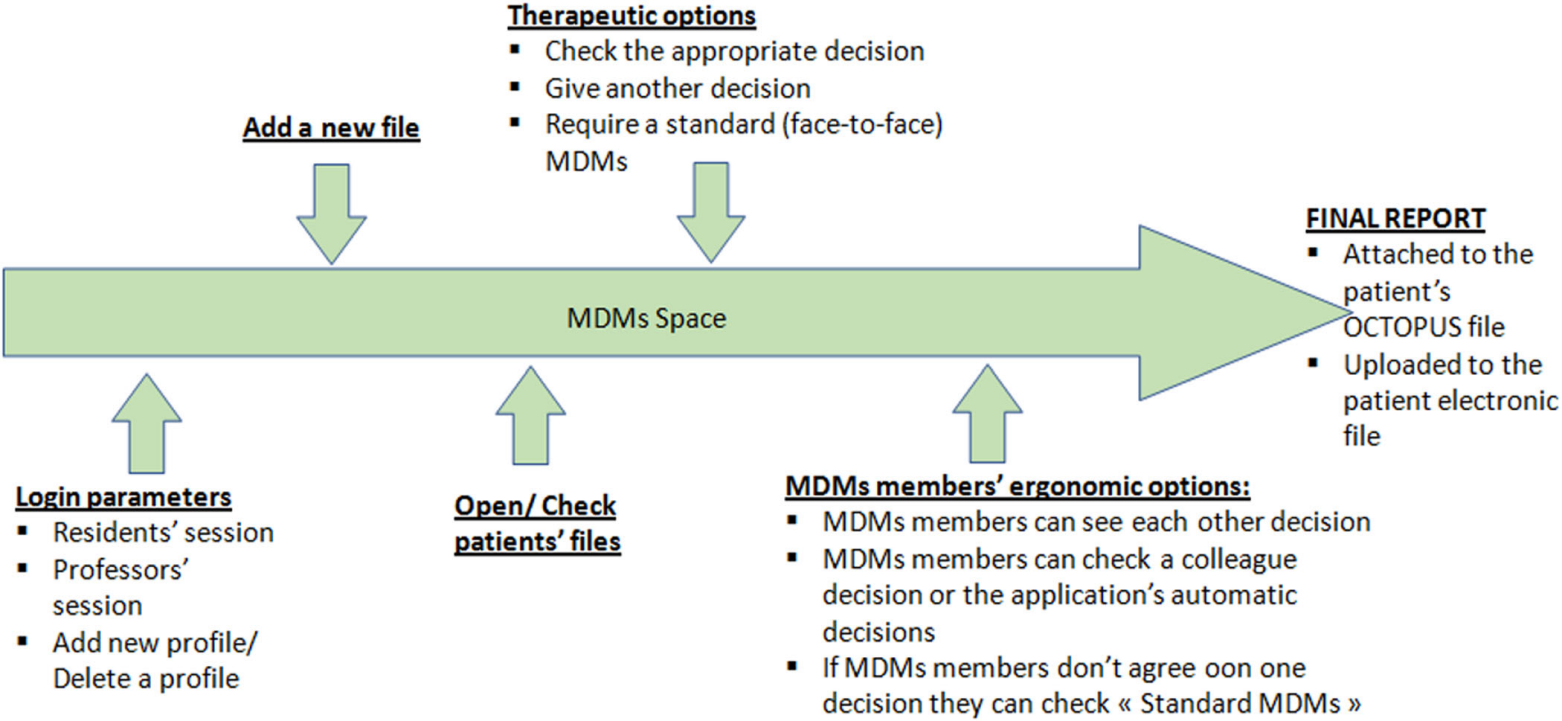
OCTOPUS overall conception chart. MDM, multidisciplinary team meetings.

The overall design plan of the application is reported in Figure 1.

□ *Identification interfaces*:
  At our university hospital, we suggested 2 types of login sessions, resident session and professor/consultant session.
  – *Resident session*: is a personal session where residents can add MDMs patients' files to the list for discussion. They fill in the required data that summarizes the patient's case.
  – *Professor/consultant session*: In our hospital, the ORL MDMs team includes.
  Otolaryngologist, an oncologist, a radiotherapist, a radiologist, and a pathologist. Each one has a personal session.
□ *MDMs workspace*:
  OCTOPUS offers different options depending on the user profile.
  – Professors log in to check the MDMs patients' list. Each file has to be reviewed in no longer than 15 days. We chose a deadline of fifteen days to match, at worst, the face‐to‐face MDMs that are carried out every 2 weeks in our institution.
  – Professors can check patients' recapitulative charts and the treatment options proposed by the application for each case.
  – When residents log in, they can add new files, delete or edit a file as long as it has not been submitted to the MDMs.
  – The resident can also consult the final report and attach it to the patient's electronic record.
  – Residents are allowed to see only their patients' final reports.
□ *Decisions*:
  Professors/consultants can:
  – Choose a therapeutic decision from what the application suggests.
  – Propose another “more suitable” therapeutic decision. The latter can be seen by other members and can be “checked” as the therapeutic decision evenly as all decisions suggested by the application.
  – Choose the option that the case should be discussed in a face‐to‐face MDMs.
  – If a unanimous decision cannot be reached, OCTOPUS displays the option of “face‐to‐face MDMs” in the final report.
□ *The internal mailing system:* The internal mailing system offers the possibility of requesting a special MDM member opinion, such as reviewing a patient's imaging or pathology results or soliciting a local expert opinion.

## Results

### Display of Cases' Data and Decision Interface

**Figure 2:**
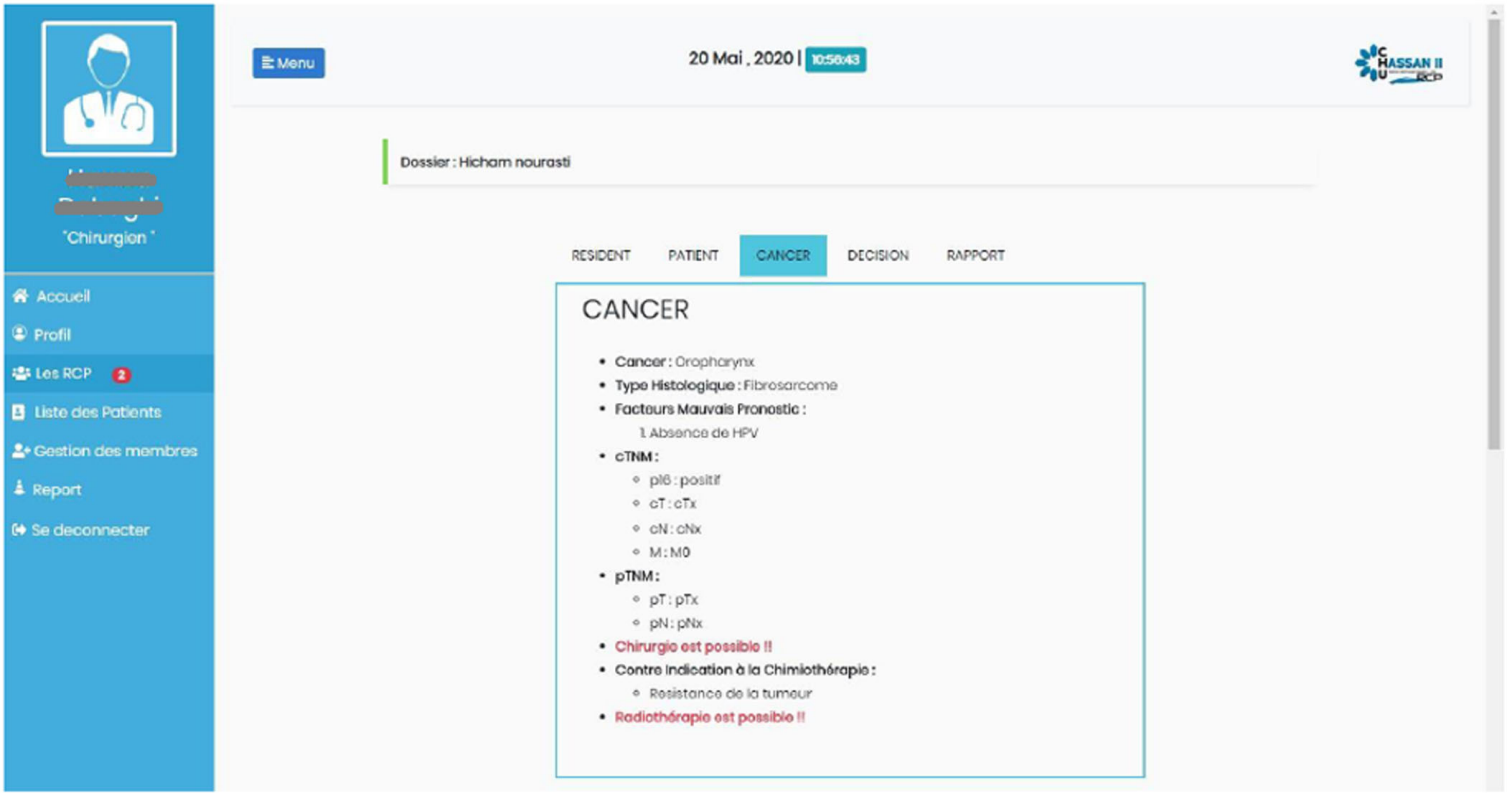
Summary review of the patient's data that includes necessary details for making an adequate decision. MDM, multidisciplinary team meetings.

Octopus displays the patient list and a summary review of each patient's data that includes the necessary details for making an adequate decision (Figure 2).

**Figure 3:**
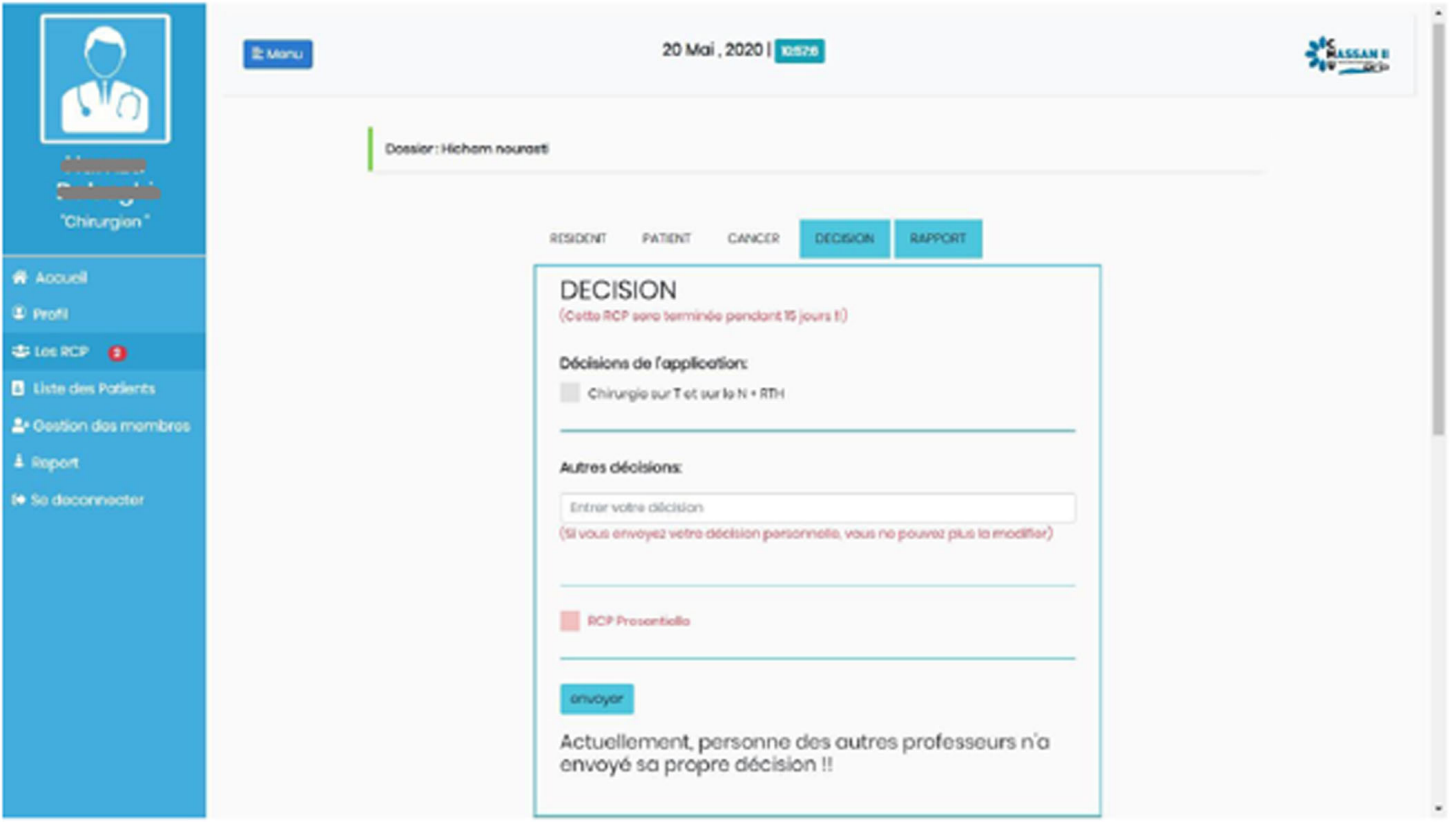
Decisions' interface before choosing a therapeutic option.

Figure 3 shows the decision interface before making a therapeutic decision.

Supporting Information: Data displays an example of a cancer management chart according to NCCN guidelines, as well as the settings and interfaces of the application.

### Cases Testing

Table 1 shows therapeutic decisions made by an expert and those proposed by the application for 10 fictitious cases. The agreement is evaluated using the *κ* coefficient. The expert was blinded regarding the automatic decision.

**Table 1:** Agreement Between Automatic and Expert's Therapeutic Decision for Fictious Cases

Case	Automatic decision	Expert decision
Larynx glottis T1N0M0 ‐ Epidermoid carcinoma ‐ Radiation: feasible ‐ Chemotherapy: feasible ‐ Surgery: feasible	Radiation or surgery	Radiation or surgery
Cavum T2N0M0 ‐ UCNT ‐ Radiation: feasible ‐ Chemotherapy: feasible ‐ Surgery: feasible	Radiation and chemotherapy	Radiation and chemotherapy
Anterior tongue T2N0M0 ‐ Epidermoid carcinoma ‐ Radiation: feasible ‐ Chemotherapy: feasible ‐ Surgery feasible	Surgery or radiation	Surgery and radiation
Oropharynx T3N1M0 ‐ Epidermoid Carcinoma ‐ Radiation: not feasible ‐ Chemotherapy: feasible ‐ Surgery feasible	Face‐to‐face MDMs	Face‐to‐face MDMs
Tongue Base T4N2cM1 ‐ Epidermoid carcinoma ‐ Radiation: feasible ‐ Chemotherapy: not feasible ‐ Surgery: not feasible	Face‐to‐face MDM	Face‐to‐face MDM
Larynx supraglottic T2N0M0 ‐ Epidermoid carcinoma ‐ Radiation: feasible ‐ Chemotherapy: feasible ‐ Surgery: feasible	Surgery + radiation or radiation	Surgery ± radiation
Mouth floor T3N1M0 ‐ Epidermoid carcinoma ‐ Radiation feasible ‐ Chemotherapy feasible ‐ Surgery: not feasible	Radiation + chemotherapy	Radiation + chemotherapy
Oropharynx T2N0M0 ‐ Epidermoid carcinoma ‐ Radiation feasible ‐ Chemotherapy: feasible ‐ Surgery: feasible	Surgery + radiation or radiation + chemotherapy	Surgery + radiation
Cavum T3N2M0 ‐ UCNT ‐ Radiation: feasible ‐ Chemotherapy: feasible ‐ Surgery: nonfeasible	Radiation + chemotherapy	Radiation + chemotherapy
Hypopharynx T3N2cM0 ‐ Relapse ‐ Radiation: not possible ‐ Chemotherapy: possible ‐ Surgery: not possible	Face‐to‐face MDM	Face‐to‐face MDM

*κ* coefficient > 0.6 (substantial agreement).

Abbreviations: MDM, multidisciplinary team meeting; TNM, tumor, node, metastasis classification.

## Discussion

There is a general agreement on the problems encountered in traditional MDM settings. The outbreak of COVID‐19 highlighted the urgency of rethinking many medical practices for the sake of efficiency and safety in a global trend toward digitalization and telemedicine for better quality and cost management.^3^, ^4^ A study suggested categorizing patients' cases into 2 groups: routine cancer cases and more complex cancer cases, which would benefit most from MDMs.^5^ We propose a semiautomatic tool for faster and more efficient management of MDM meetings with a more uniform selection of “complex cases” for face‐to‐face meetings. This tool is adaptable for all oncology disciplines. Although this is a pilot study, preliminary results are promising. However, further testing using real‐world cases and larger sample sizes is needed.

## Authors Contributions

**Naouar Ouattassi**, designed the study, established therapeutic algorithms, and drafted the manuscript; **Anas Azdad**, **Hamza Dabaghi**, and **Arsalane Zarghili**, developed the application; **Mohamed Nouredine El Amine El Alami**, designed the study and reviewed the manuscript for significant insights.

## Disclosures

### Competing interests

The software used for the development of this application is the original version (with license) belonging to the computer engineering department of the Faculty of Science and Technology, University Sidi Mohammed BenAbdellah, Fez, Morocco.

### Funding source

Not applicable.

## Supporting information

Supplemental data: displays an example of a cancer management chart according to NCCN guidelines, as well as the settings and interfaces of the application.
