# Supplementary material for: “OCTOPUS”: An Intelligent Tool for Assisted Multidisciplinary ORL Oncology Meetings—Preliminary Study
Source: OTO Open. 2023 Jul 12;7(3):e64. doi: 10.1002/oto2.64 (PMC10336480; doi:10.1002/oto2.64)
Supplement: Supplementary file 1 — Supplemental data: displays an example of a cancer management chart according to NCCN guidelines, as well as the settings and interfaces of the application. [file OTO2-7-e64-s001.pptx]

## Slide 1
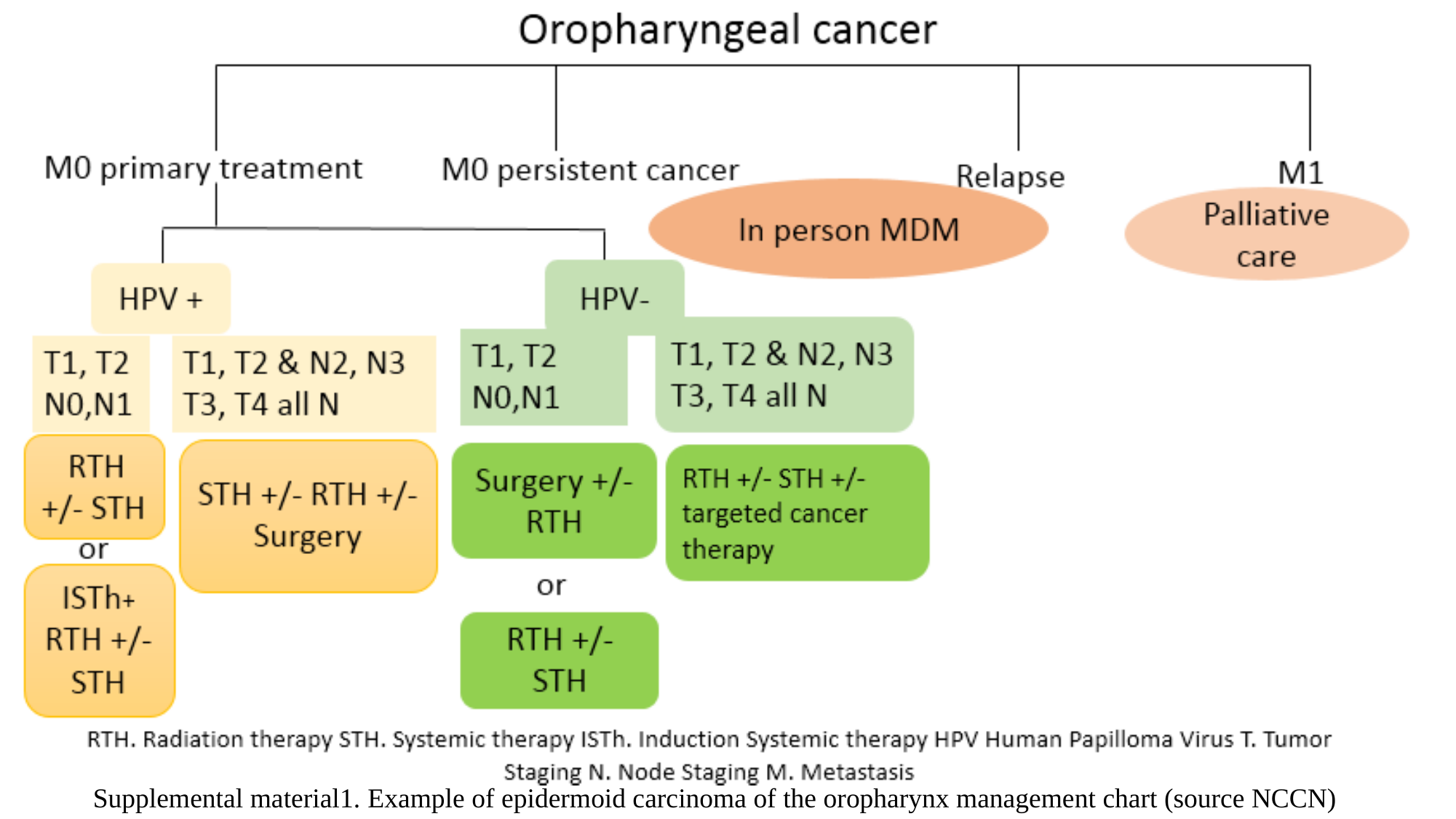

Supplemental material1. Example of epidermoid carcinoma of the oropharynx management chart (source NCCN)

## Slide 2
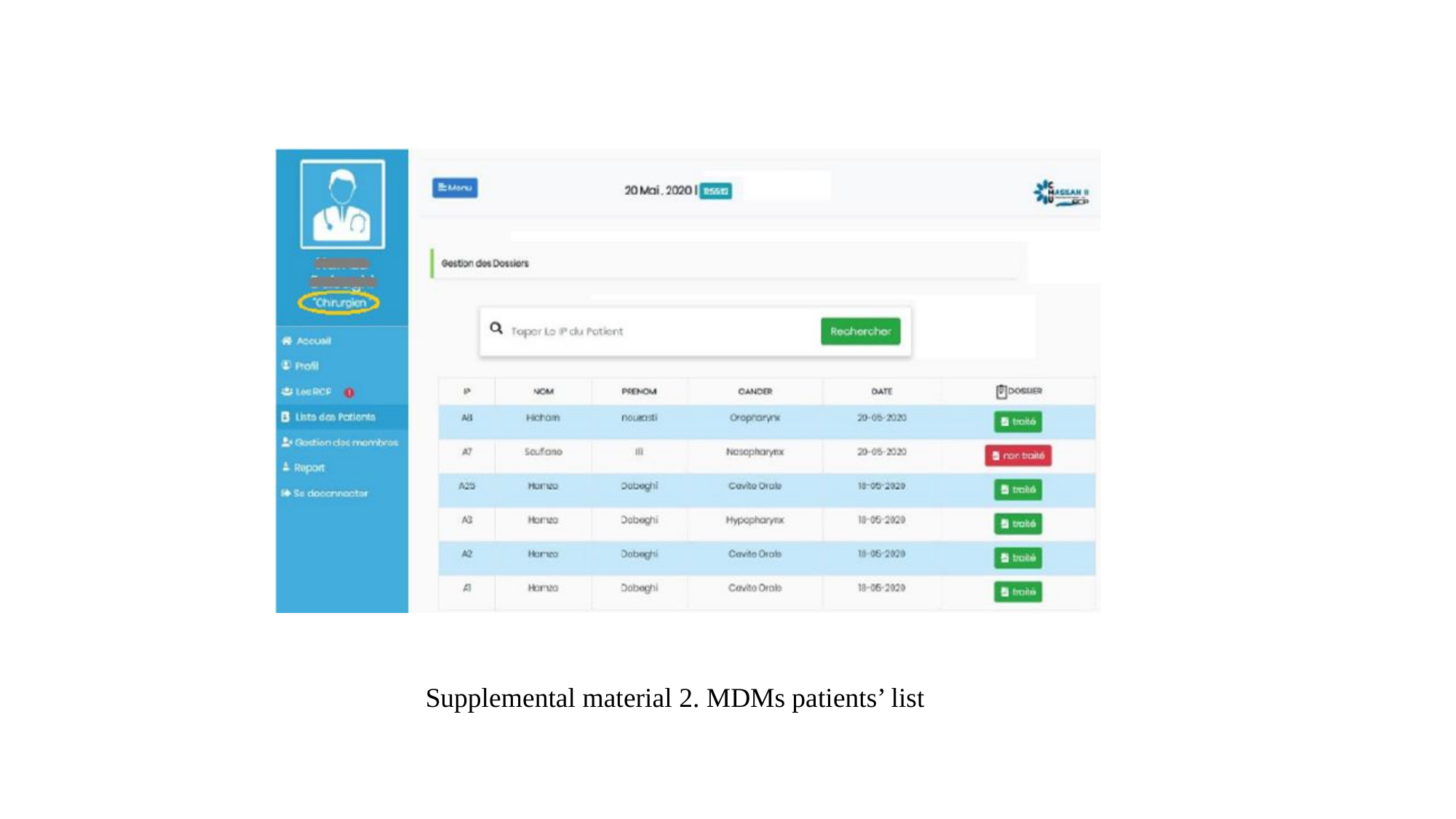

Supplemental material 2. MDMs patients’ list

## Slide 3
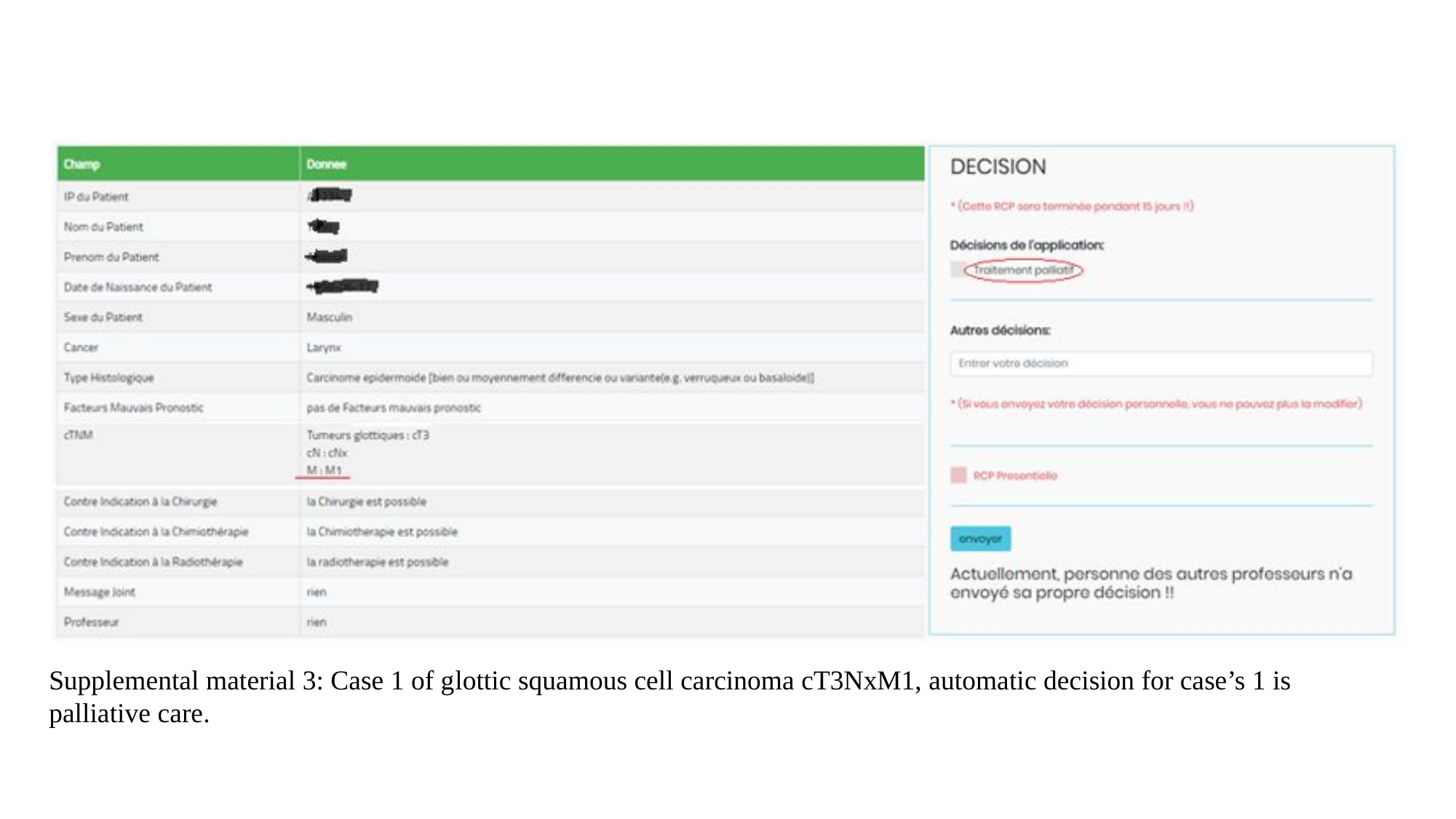

Supplemental material 3: Case 1 of glottic squamous cell carcinoma cT3NxM1, automatic decision for case’s 1 is palliative care.

## Slide 4
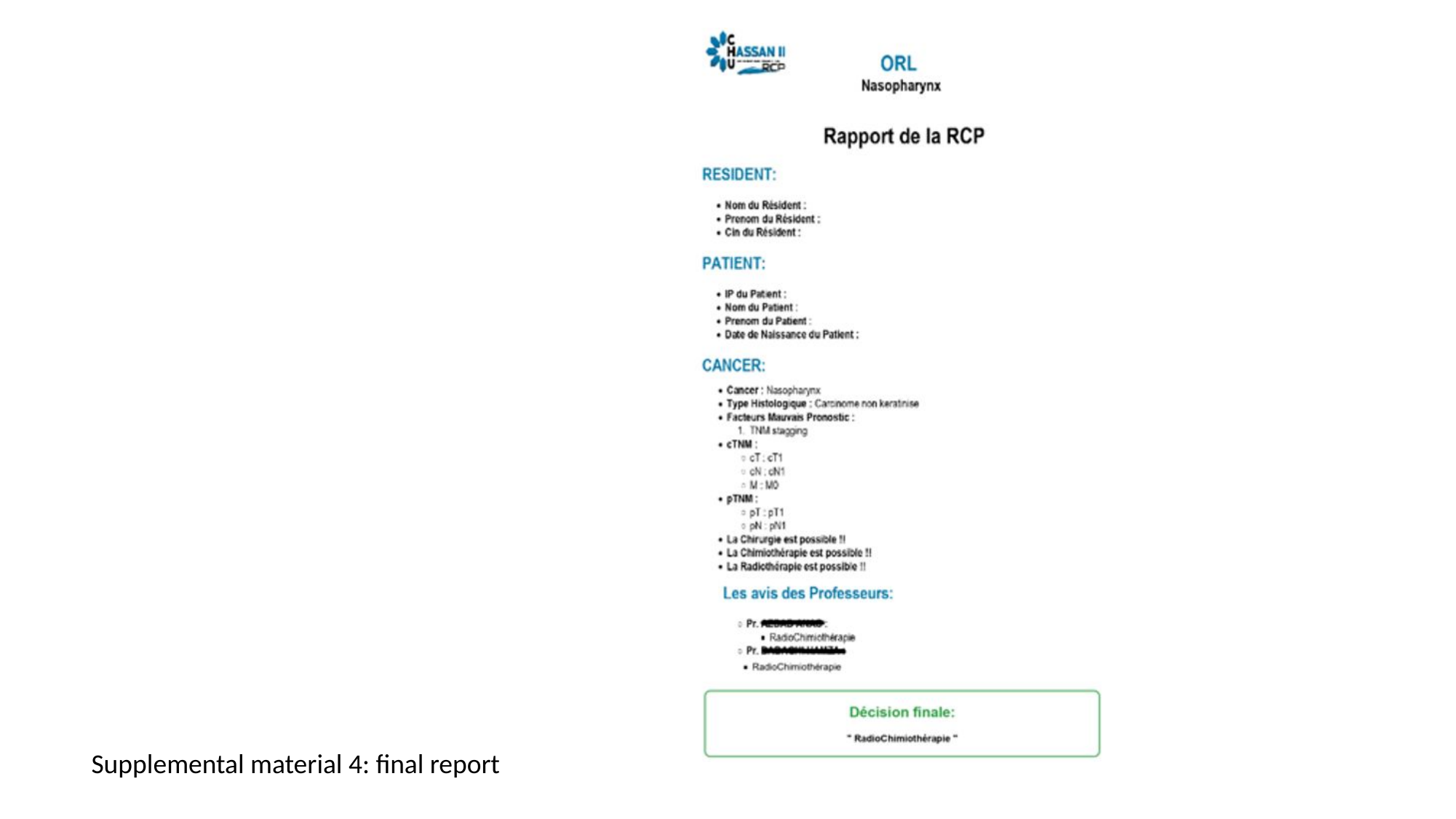

Supplemental material 4: final report
